# Combined Experimental and Computational Study on the Structure–Property Relationships of Mono- and Dicationic Imidazolium Ionic Liquids for CO_2_ Capture

**DOI:** 10.1021/acs.jpcb.5c07289

**Published:** 2026-03-10

**Authors:** Evandro Duarte, Vitor Forneck, Everton Motta, Leonardo dos Santos, Nadezhda A. Andreeva, Vitaly V. Chaban, Franciele L. Bernard, Sandra Einloft

**Affiliations:** † Graduate Program in Materials Engineering and Technology-PGETEMA, Pontifical Catholic University of Rio Grande do Sul-PUCRS, Porto Alegre, Rio Grande do Sul 90619-900, Brazil; ‡ School of Technology, Pontifical Catholic University of Rio Grande do Sul-PUCRS, Porto Alegre, Rio Grande do Sul 90619-900, Brazil; § Peter the Great St. Petersburg Polytechnic University, St Petersburg 195251, Russian Federation; ∥ Yerevan State University, Yerevan 0025, Armenia

## Abstract

The present study investigates the potential of dicationic ionic liquids (DILs) and monocationic ionic liquids (MoIL), with and without metal in the anion, for CO_2_ capture applications. The structures of the samples were confirmed by FTIR, ^1^H NMR spectroscopy, and Raman spectroscopy, while their physicochemical properties, density, viscosity, and thermal stability were evaluated. A series of computational simulations were conducted by using density functional theory (M11/def2-TZVP) to ascertain the multiplicity of the ground state of the magnetic anion [FeCl_4_]^−^. These simulations determined the multiplicity to be a sextet and furthermore identified the trans conformation as the most energetically favorable for cation [E­(MIM)_2_]^2+^. This finding demonstrates a correlation between the structural conformations and the experimental Raman spectra. The findings of CO_2_ sorption and kinetic tests, conducted under postcombustion conditions (40 °C, 4 bar), indicated that DILs exhibited superior performance in comparison to MoILs. The DIL [E­(MIM)_2_]­[2Cl] exhibited the highest sorption capacity (110.20 μmol/g), which is almost three times higher than that of the best MoIL (BMIM FeCl_4_). These enhancements can be ascribed to reduced viscosities and an augmented number of active interaction sites in the dicationic structures. Furthermore, [E­(MIM)_2_]­[2Cl] exhibited a high degree of selectivity for CO_2_ over N_2_ and demonstrated stability over five recycling cycles, suggesting the potential of DILs as candidates for the development of CO_2_ capture technologies.

## Graphical abstract

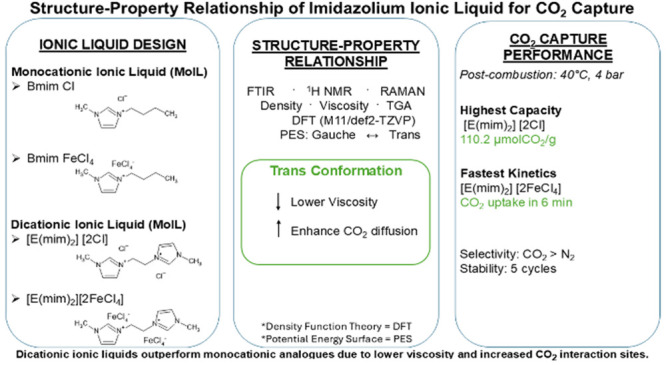

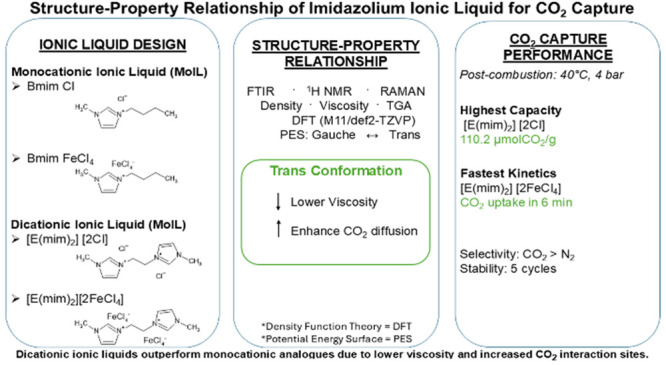

## 1 Introduction

The rise in global temperatures has sparked considerable concern and has become a central topic of political and academic debate worldwide. The primary objective is to limit the temperature increase to less than 2 °C compared to the preindustrial period to mitigate the adverse impacts on our planet. The increasing frequency and intensity of extreme natural events associated with rising temperatures further emphasize the urgency of this issue.^1^

The increase in temperature is mainly linked to the intensification of the greenhouse effect, driven by the excessive emission of the main gases resulting from human activities, especially the burning of fossil fuels such as coal, oil, and natural gas, by the industrial and energy sectors. The main greenhouse gases include carbon dioxide (CO_2_), methane (CH_4_), nitrous oxide (N_2_O), and fluorinated gases. CO_2_ stands out as the most significant contributor, accounting for approximately 75% of total emissions and having a longer residence time in the atmosphere.^2^ Studies show that CO_2_ is responsible for two-thirds of the energy imbalance on the planet.^3^

One method to combat climate change involves the mitigation of CO_2_ through carbon capture and storage (CCS) technology. This technology entails the selective capture of CO_2_, followed by its compression to a supercritical state, transportation, and storage in geological formations. The most common geological storage sites are deep saline aquifers, coal deposits, and depleted oil/gas fields.^4^ The CO_2_ capture process can be carried out basically by three types of systems: Precombustion, which occurs through conversion of fossil fuel into gas and removing CO_2_ before combustion. Oxi-combustion, which uses high-purity oxygen (>95%) for fossil fuel burning to generate a gas mixture of water vapor and CO_2_ ready for capture; and postcombustion, capturing CO_2_ after the fossil fuel burning. Postcombustion is the most widely used system among the three due to its minimal impact on existing infrastructures.^5,6^

The most common CO_2_ capture method utilizes selective solvents with amine solutions being the most widely used. These solvents are diluted in water and perform chemical absorption at low pressure and temperature, typically ranging from 30 to 50 °C. The captured CO_2_ is then removed at a high temperature (about 120 °C) in a regeneration reactor before being condensed for transport and storage.^7,8^

Amines are widely used due to their low cost, high absorption capacity, and fast reaction kinetics with CO_2_.^9^ However, drawbacks include the substantial energy requirements for the solvent regeneration process, equipment corrosion, and high degradability of the absorbents, which can increase process costs.^6^ In order to address these issues, a number of materials have been evaluated as potential replacements for aqueous amine solutions, including mesoporous materials, membranes, composite materials, and others.^10,11^ Among them are the Ionic Liquids (ILs), which are salts composed of organic cations and anions that can be organic or inorganic, that remain liquid at temperatures below 100 °C, and have their properties tailored according to cation and anion combinations.^12^

ILs offer several advantages over aqueous amine solutions, such as negligible vapor pressure, low corrosivity, high thermal stability, adjustable structure, and recyclability.^13,14^ However, their high viscosity makes the absorption process difficult, resulting in lower performance compared to aqueous amine solutions. To mitigate this issue, small quantities of IL can be used within mesoporous or membrane materials, increasing the selectivity of the material for CO_2_.^15,16^

Novel ILs, exhibiting notably improved CO_2_ capture capacity compared to traditional ones, have undergone assessment in this study, including dicationic ionic liquids (DILs) and magnetic ionic liquids (MILs). The DILs are a class composed of two cations, bound by a ligand that can be of different chain types, and two anions. These were insufficiently studied in the CO_2_ capture aspect compared to the monocationic ionic liquid (MoIL) data. An increase in the number of interaction sites in DIL compared to monocationic appears to be beneficial for interactions with CO_2_ molecules.^17,18^ MIL is characterized by the addition of a transition metal in the anions or cations, which can be influenced by an external magnetic field altering the viscosity, solubility, and solvating ability to other chemical compounds, generating interest in CO_2_ capture processes.^19^

In this study, MoILs and DILs, magnetic and nonmagnetic, were synthesized, and the physicochemical properties, CO_2_ capture capacity, and kinetics were presented. To provide practical data regarding these materials in their pure state, the tests were conducted under conditions similar to those of a postcombustion system at 40 °C. Furthermore, computational simulation studies were employed to determine the natural multiplicity, perform potential energy surface scans, and calculate Raman spectra, which were compared and discussed with the experimental spectra for validation purposes.

## 2 Materials and Methods

### 2.1 Materials

1-Methylimidazole (≥99.0%, Sigma-Aldrich, Germany), 1,2-dicholoethane (≥99.0%, Synth, Brazil), 1-chlorobutane (≥99.5%, Sigma-Aldrich, USA), iron­(III) chloride (FeCl_3_, ≥97.0%, Sigma-Aldrich, Germany), toluene anhydrous (≥99.8%, Sigma-Aldrich, USA), dichloromethane (CH_2_Cl_2_, P.A., Synth, Brazil), and acetonitrile (CH_3_CN, ≥99.0%, Merck, USA).

### 2.2 Synthesis of the 1-Butyl-3-methylimidazolium Chloride (BMIM Cl)

The synthesis was performed according to methods reported in the literature^20,21^ with specific modifications aimed at improving the efficiency of the reaction and the purity of the product. To synthesize BMIM Cl, illustrated in Figure 1, 1-methylimidazole and 1-chlorobutane were added in a molar ratio of 1:1.5. The mixture was stirred for 24 h under a nitrogen atmosphere at 70 °C. Subsequently, the synthesized BMIM Cl was precipitated dropwise in dry toluene at 0 °C, resulting in the formation of white crystals. The toluene was then removed under vacuum to obtain the final product (yield of 86%).

**Figure 1:**
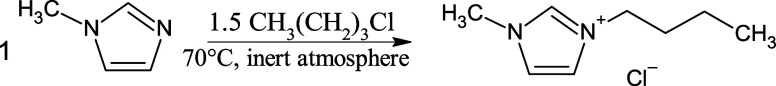
Synthesis of the BMIM Cl.

### 2.3 Synthesis of the 1-Butyl-3-methylimidazolium Tetrachloroferrate (III) (BMIM FeCl_4_)

The synthesis of BMIM FeCl_4_, illustrated in Figure 2, was carried out based on prior methodologies.^22,23^ BMIM Cl, obtained from the previous reaction, underwent the addition of the FeCl_3_ salt in a molar ratio of 1:1.1. The mixture was stirred for 3 h in an inert atmosphere at room temperature. Subsequently, the BMIM FeCl_4_ was treated with dry acetonitrile and stirred for 30 min to remove larger unreacted iron particles. The removal process was facilitated under vacuum at 50 °C, obtaining a homogeneous ionic liquid (yield of 79%).

**Figure 2:**
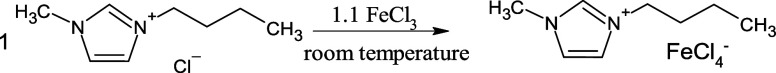
Synthesis of BMIM FeCl_4_.

### 2.4 Synthesis of the 3,3′-(Ethane-1,2-diyl)-bis­(1-methylimidazolium)­bis Dichloride {[E­(MIM)_2_]­[2Cl]}

Following methods reported in the literature^24,25^ with appropriate modifications, for the synthesis of [E­(MIM)_2_]­[2Cl], illustrated in Figure 3, in a three-necked flask, 1-methylimidazole and 1,2-dichloroethane were introduced in a molar ratio of 2.1:1, with dry acetonitrile serving as the reaction solvent. The reaction mixture remained in a reflux system for 24 h. Afterward, acetonitrile was removed under a vacuum at 50 °C. Dry acetonitrile was then added to the reaction flask to remove unreacted species, and the evacuation process was repeated at 50 °C (yield of 88%).

**Figure 3:**
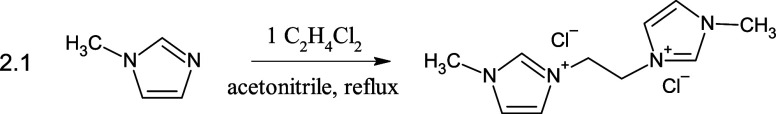
Synthesis of [E­(MIM)_2_]­[2Cl].

### 2.5 Synthesis of the 3,3′-(Ethane-1,2-diyl)-bis­(1-methylimidazolium)-bis­[tetrachloroferrate (III)] {[E­(MIM)_2_] 2FeCl_4_}

[E­(MIM)_2_]­[2FeCl_4_], illustrated in Figure 4, was synthesized based on established procedures.^26,27^ It was obtained by the addition of FeCl_3_ to [E­(MIM)_2_]­[2Cl] in a molar ratio of 2.1:1. The reaction mixture was stirred for 3 h in a three-necked flask under an inert atmosphere at room temperature. Dry acetonitrile was then added and left to stir for 10 min, followed by vacuum to remove unreacted iron particles (yield of 73%).

**Figure 4:**
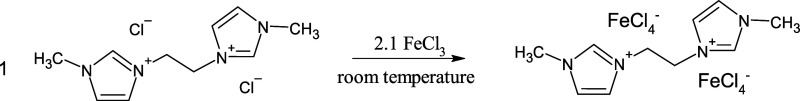
Synthesis of [E­(MIM)_2_] and [2FeCl_4_].

### 2.6 Characterization

The ILs were synthesized, and their structures were confirmed by Fourier transform infrared (FTIR) spectroscopy using a PerkinElmer Spectrum Three equipped with a Universal Attenuated Total Reflectance sensor (UATR-FTIR). Proton nuclear magnetic resonance (1H NMR) analyses in the liquid state were performed using a Bruker Advance DRX-400 spectrometer operating at 400 MHz. Five mg of IL was added to 1 mL of D_2_O in 5 mm NMR glass tubes for analysis. Raman spectroscopy was performed using a Horiba LabRamHR evolution laser Raman spectrometer, model DXR (laser excitation wavelength of 532 nm), and Access alpha was used 300 (632.8 nm – micro-Raman single-spot analysis and mapping microscope). In the computer simulation, the determination of natural multiplicity, the potential energy surface scans, and the calculations of Raman spectra were conducted using the capabilities of the Gaussian 09 package.1 Avogadro 1.2 was used to build initial geometries and specified variables that control simulations.^28^ Lone ions and ion pairs were used to represent the ionic liquids. Note that computational studies such as the ones reported herein are based on simplified atomistic compositions and neglect long-range interatomic interactions. The geometry optimization jobs were performed to obtain force-free ionic structures using the rational function optimization (RFO) algorithm at the unrestricted M11/def2-TZVP level of theory. M11 is a hybrid meta-GGA density functional theory method.^29^ M11 includes dispersion corrections^30^ on the fly, which is paramount to reliably simulating noncovalent interaction energies between the studied cations and anions. M11 was parametrized versus experimental data on organic compounds as described in the method derivation publication by Peverati and Truhlar.^29^ The def2-TZVP is a large triple-ζ atom-centered polarized basis set,^31^ which we extensively applied earlier to similar liquid ionic structures. The physicochemical properties of the ILs were analyzed, with density determined using a Gay-Lussac glass pycnometer, at a temperature of ∼40 °C. Viscosity measurements were conducted using a Brookfield viscometer DV-I prime, along with a thermostatic bath, to maintain the analysis temperature at 40 °C. Thermogravimetric analysis (TGA) was carried out to determine the ILs degradation temperatures and potential moisture content, in the temperature range from 25 to 600 °C, in a nitrogen atmosphere, utilizing the SDT Q600 V20.9 Build 20 equipment. The CO_2_ sorption and kinetic tests were carried out in a constant volume equilibrium cell at an intermediate temperature of 40 °C within the typical range of 30–50 °C for postcombustion systems. The experiments were conducted at an equilibrium pressure of 4 bar over a 60 min period, following a vacuum drying process for the ILs for 1 h, in a system similar to that described by Jacquemin et al.^32^ In the ionic liquid with the best performance for CO_2_ capture, the test was performed with nitrogen (N_2_) by following the same procedure to evaluate the selectivity between the two gases. Finally, a 5-cycle recycling test was performed under the same conditions as the CO_2_ sorption test on the best-performing sample.

## 3 Results and Discussion

### 3.1 Analysis of Chemical Structures

The FTIR spectra of the studied ILs are depicted in Figure 5. Notably, the spectra exhibit significant similarities, which can be attributed to the analogous functional groups shared among the different ILs. The band appearing at approximately 3400 cm^–1^ is attributed to strong OH stretching vibrations, typically associated with moisture being absorbed by the ILs. Bands in the range of 3150–3050 cm^–1^ are attributed to the CH stretching of the imidazole ring.^33^ The bands at 2950 cm^–1^ and 2870 cm^–1^ are specific to asymmetric and symmetric aliphatic stretching of the methyl (CH_3_) and methylene (CH_2_) groups, respectively. Vibrations in the regions around 1650, 1560, and 1460 cm^–1^ correspond to the functional groups in the imidazole ring, specifically C=N, C=C, and C–N.^34^ The strong absorption band near 1160 cm^–1^ is related to vibrations of the aliphatic C–N bond and the C–C alkyl chain of the cations.^35,36^ Additionally, the band around 745 cm^–1^ is characteristic of the out-of-plane C–H bending vibration of the imidazole ring.^37^

**Figure 5:**
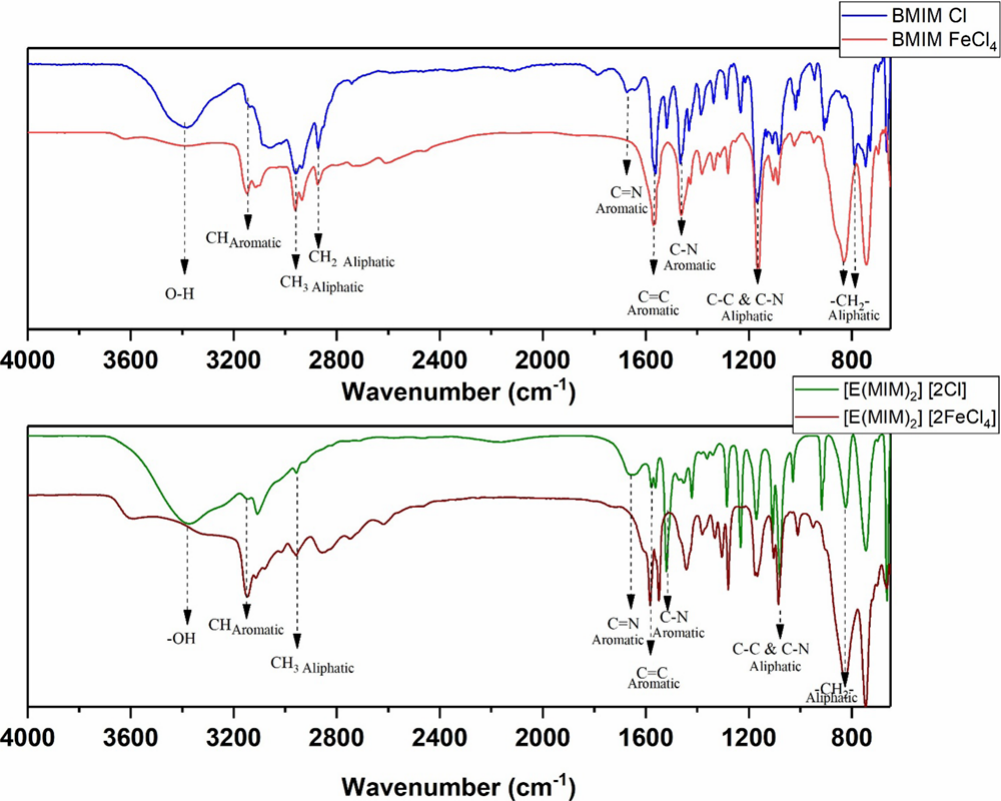
FTIR spectrum of BMIM Cl, BMIM FeCl_4_, [E­(MIM)_2_]­[2Cl], and [E­(MIM)_2_]­[2 FeCl_4_].

^1^H NMR spectroscopy was employed to determine the chemical structure of the cations evaluated in this study. However, the incorporation of iron into the ILs rendered them paramagnetic, making NMR spectroscopy impractical. According to the literature, this paramagnetism broadens spectral peaks and hampers the ability of spectrometers to lock onto the deuterium signal. Consequently, only the structures of BMIM Cl and [E­(MIM)_2_]­[2Cl] were analyzed. The presence of the anions (FeCl_4_^–^) was confirmed using Raman spectroscopy, as supported by prior studies.^38,39^

As shown in Figure 6, the chemical shifts observed in the ^1^H NMR spectra facilitated the identification of structural differences between the cations, complementing the information obtained from the FTIR spectra. For BMIM Cl (Figure 6a), distinct peaks were observed at 9.75 ppm (−N–CH^8^=N−), 7.99 and 7.90 ppm (−N–CH^7^=CH^6^–N−), 4.23 ppm (−N–CH_2_^5^–CH_2_−), 3.90 ppm (−N–CH_3_^4^−), 1.75, 1.21, and 0.84 ppm (CH_2_^3^–CH_2_^2^–CH_3_^1^). These findings aligned with previously reported characterizations in the literature.^40,41^ Similarly, the ^1^H NMR spectrum of [E­(MIM)_2_] 2Cl (Figure 6b) exhibited peaks at 7.33 ppm (−N–CH^5^=N−), 6.95 and 6.80 ppm (−N–CH^3^=CH^4^–N−), 3.60 ppm (−N–CH_3_^2^−), and −0.07 ppm (−CH_2_^1^−), which are consistent with reported values.^25^

**Figure 6:**
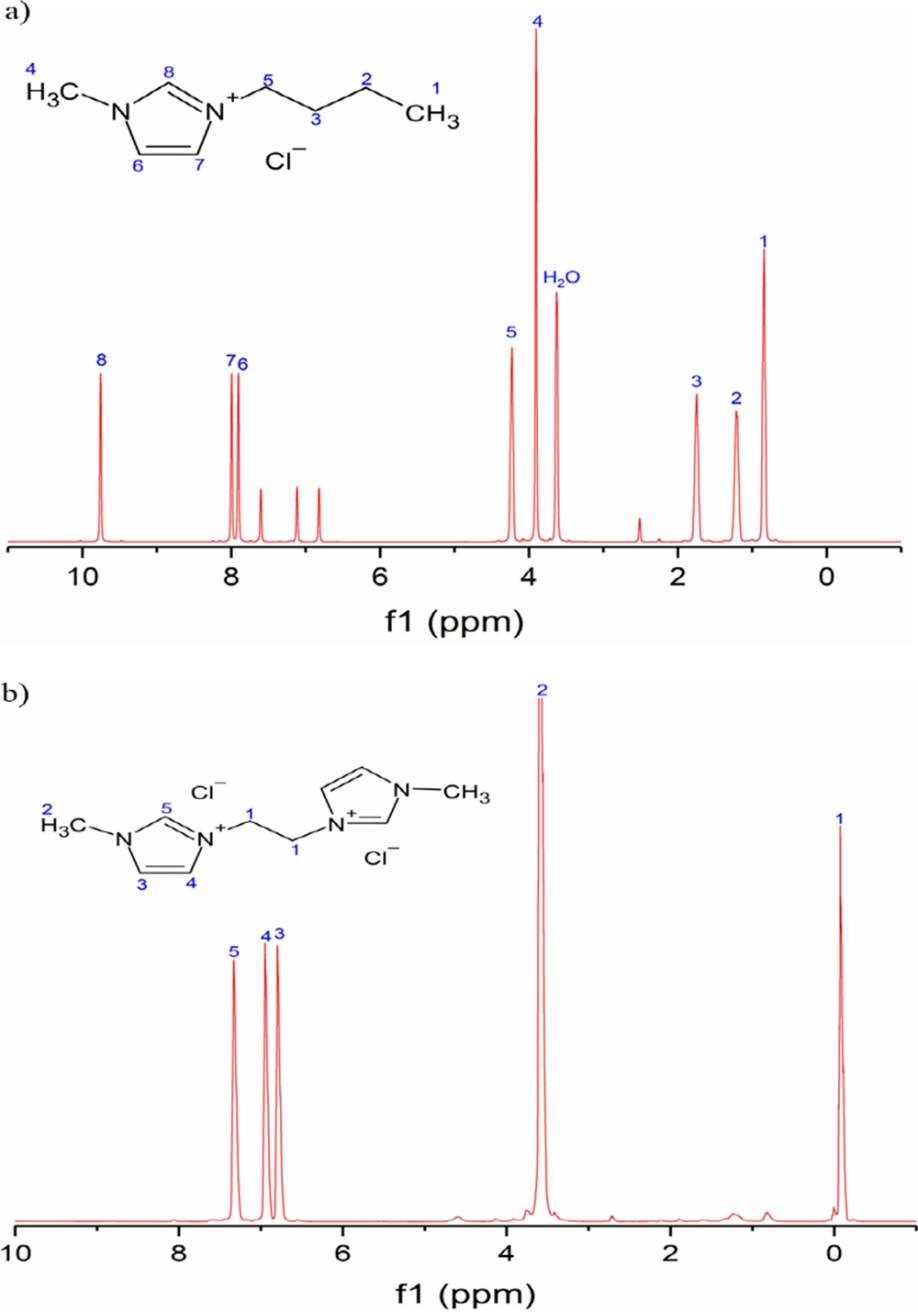
^1^H NMR spectra of (a) BMIM Cl and (b) [E­(MIM)_2_]­[2Cl].

A comparison of the Raman spectra for BMIM Cl and [E­(MIM)_2_]­[2Cl], alongside the spectra of BMIM FeCl_4_ and [E­(MIM)_2_]­[2FeCl_4_], is presented in Figure 7. The ILs containing FeCl_4_^–^ anion showed a strong band at 336 cm^–1^, corresponding to fully symmetric Fe–Cl stretching. This observation supports the successful formation of BMIM FeCl_4_ and [E­(MIM)_2_]­[2FeCl_4_].^42,43^

**Figure 7:**
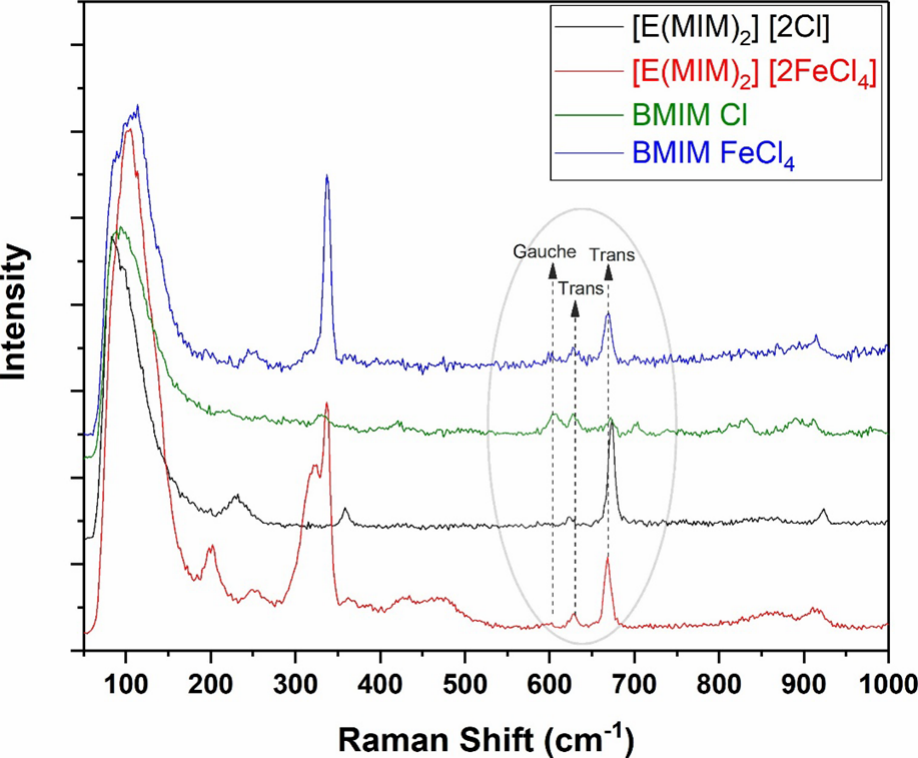
Raman spectra of [E­(MIM)_2_]­[2Cl], [E­(MIM)_2_]­[2FeCl_4_], BMIM Cl, and BMIM FeCl_4_.

Raman analysis also provides insight into cation structures. Peaks observed in the 600–700 cm^–1^ region shed light on the conformations of gauche (around 608 cm^–1^) and trans (around 630 cm^–1^) isomers. These peaks are associated with symmetrical deformational vibrations of the imidazole ring, as well as C–N stretching in the branched *n*-butyl groups of MoILs, the n-ethyl groups of DILs, and the methyl groups of 1-methylimidazole.^44–46^ BMIM Cl, existing as a solid at room temperature, exhibits coexisting gauche and trans conformations with a slight dominance of gauche. In contrast, the other ILs, which are molten at room temperature, predominantly present the trans conformation. This behavior is attributed to the gauche conformation, which involves stronger interactions between the cation and the anion, with the halide anion (Cl^–^) being closer to the cation.^47,48^

Notably, the intensity of the trans conformation peaks (around 670 cm^–1^), associated with asymmetric deformational vibrations of the imidazolium ring coupled to the C–N stretching of the *n*-butyl groups in MoILs, the n-ethyl groups of DILs, and the methyl groups of 1-methylimidazole, is exclusive to molten-state ILs at room temperature.^49,50^ The data in Table 1 corroborate these findings, indicating an inverse relationship between the intensity of the trans-conformation peaks (around 670 cm^–1^) and the viscosity of the ILs. Higher intensities of these peaks correspond to lower viscosities, suggesting that structural conformation significantly influences macroscopic properties.

**Table 1:** Physical–Chemical Properties of the ILs Used^a^

	MW	density	viscosity	degradation temperature		
ionic liquid	g/mol	g/cm^3^ (at 40 °C)	cP (at 40 °C)	onset (°C)	max (°C)	end (°C)
BMIM Cl	174.67	1.12	ND	269	293	306
BMIM FeCl_4_	336.87	1.40	174.5	380	416	437
[E(MIM)_2_][2Cl]	261.12	1.14	8.5	286	316	330
[E(MIM)_2_][2FeCl_4_]	585.52	1.54	53	264	319	361

a ND: not determined.

Consistent with previous studies, the positioning of the anion and cation within the ILs plays a crucial role in determining physical properties such as viscosity and melting point.^51^ The observed changes in viscosity and other macroscopic properties can be attributed to a combination of factors, including hydrogen bonding, Coulombic interactions between ions, and van der Waals repulsions among the alkyl chains of imidazolium cations. These interactions depend on the size, shape, and chemical composition of both the cations and the anions.^45,52–55^

### 3.2 Computational Section

The iron­(III) tetrachloride is a magnetic anion because of the presence of the iron core. Such systems typically exhibit a high multiplicity. To determine the most stable spin state, the system’s electronic potential energy was evaluated as a function of the FeCl_4_ anion multiplicity. Note that central Fe^3+^ contains five unpaired electrons (valence electronic configuration of d^5^), and each linked chlorine is a singlet. Consequently, possible aggregate multiplicities of the FeCl_4_ anion are two, four, and six. The results in Figure 8 unravel an energy minimum at a multiplicity of six. The multiplicity, which corresponds to the lower energy, must be considered to be a ground-state multiplicity. The lower multiplicities of the magnetic anion, two and four, appear to be substantially higher in electronic energy; particularly, the doublet state is thermodynamically unstable. Compare the energies of +62 and +243 kJ/mol in the quartet and doublet states of the FeCl_4_ anion, respectively. Furthermore, the lowest-energy system converges its self-consistent field procedure into an essentially smaller number of iterations.

**Figure 8:**
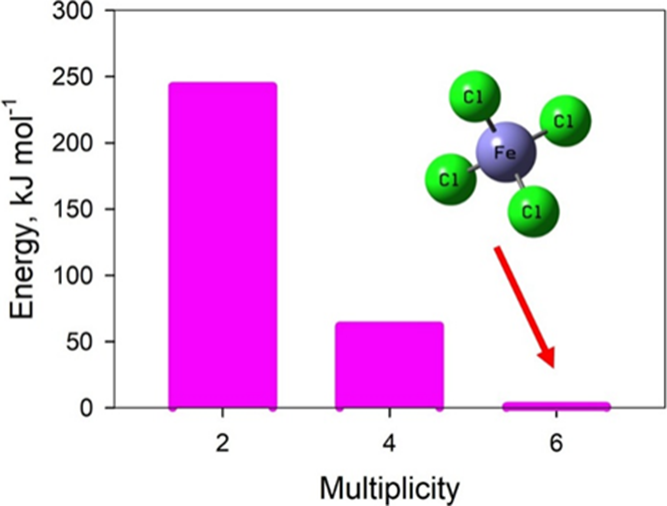
Relative electronic energy decrease of the FeCl_4_ anion upon increasing its multiplicity. The relative energy of the sextet electronic configuration is set to zero for simplicity.

Since a single anion exhibits a multiplicity of six, the ion pairs containing two such anions must exhibit a multiplicity of 11. This essential finding was used to thoughtfully set up all of the following simulations of the magnetic ion pair.

To identify stable conformations, a rotatory potential energy surface scan of the [E­(MIM)_2_] cation was performed in its central part. Specifically, the N–C–C–N dihedral angle uniting the imidazole rings was rotated stepwise by 2 degrees. The complete rotation (360°) was simulated (180 scan steps). The electronic potential energy was minimized at every step by performing partial geometry optimization using RFO until there were negligibly small atomic forces.

One of the energy minima occurs at 180° of the N–C–C–N dihedral angle. By definition, this is the trans conformation of the [E­(MIM)_2_] cation. The two remaining minima are symmetric gauche conformations. Their rotatory coordinates are 70° and 288 degrees. Both gauche conformations are +8 kJ/mol higher in potential energy as compared to the trans conformation (Figure 9). Before Raman spectra were computed, the Cl anion and FeCl_4_ anion were added to the separate gauche and trans systems. The obtained neutral systems were appropriately optimized.

**Figure 9:**
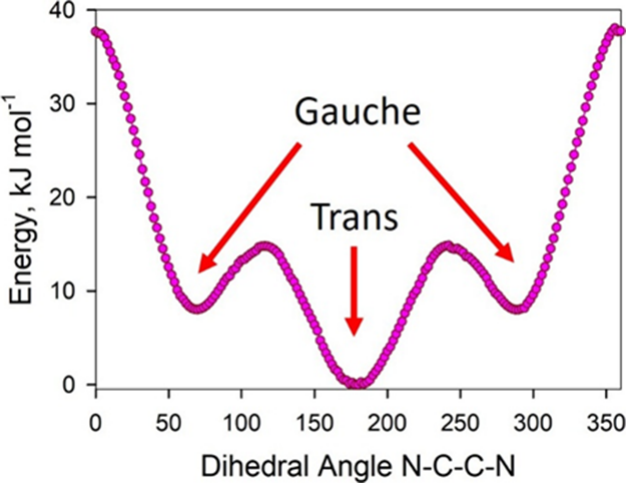
Electronic energy versus rotating dihedral angle N–C–C–N of the [E­(MIM)_2_] cation.

The Raman scattering activities must be derived from the first derivatives of the molecular polarizability tensor with respect to each normal coordinate. The Raman activity of each vibrational mode (*A_i_*) was calculated as *A_i_* = 45­(α′_*i*_)^2^ + 7­(γ′_*i*_)^2^, in which α′_*i*_ and γ′_*i*_ are the isotropic and anisotropic components of the polarizability derivative, respectively. The relative Raman intensity (*I_i_*) was obtained from the activity according to

$$I_{i}=[A_{i}\times (\nu_{0}-\nu_{i}{)}^{4}]/[\nu_{i}\times (1-\exp(-hc\nu_{i}/kT))]$$

in which ν_*i*_ is the vibrational frequency, ν_0_ is the laser excitation frequency, and *T* is the temperature. The temperature of 298 K was used in the current calculations. Simulated Raman spectra were generated from the calculated activities using Gaussian broadening (10 cm^–1^ was herein chosen to be half-width at half-height). Note that Raman spectra are harmonic by nature. For an accurate comparison with the experiment, computed frequencies must often be scaled akin to the infrared spectrum. However, we did not find the necessity to scale the obtained Raman frequencies after comparison with the recorded experimental spectrum. It is possible that systematic errors of the employed calculation algorithm were canceled to a large extent.

Figure 10 shows the calculated Raman spectra in the [E­(MIM)_2_]­[2Cl] system in the gauche and trans conformations. A characteristic peak was observed at 609 cm^–1^ for the gauche conformation. In turn, the trans conformation manifested itself at a higher frequency, specifically, 652 cm^–1^. These frequencies are in satisfactory relationship and numerical agreement with the experimental Raman spectra, as discussed above.

**Figure 10:**
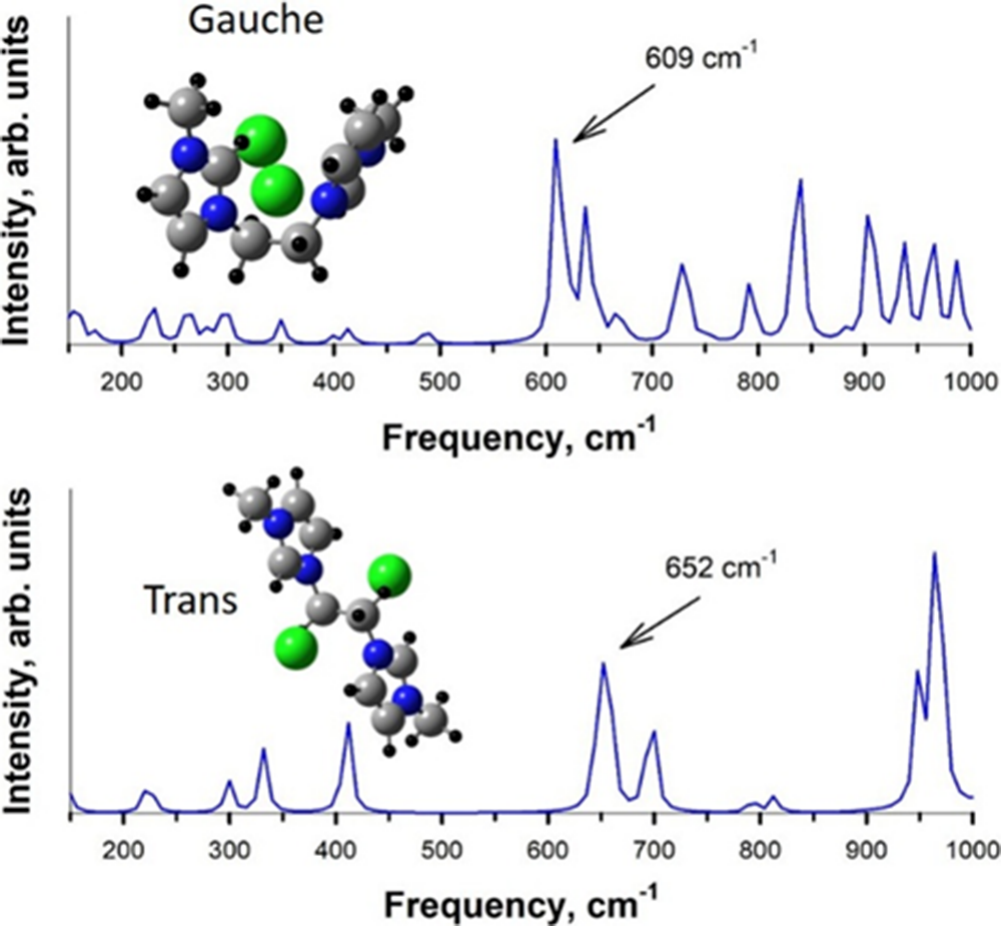
Raman spectra for [E­(MIM)_2_]­[2Cl] in the gauche conformation (top) and the trans conformation (bottom). The simulated geometries of the respective conformations are provided as insets.

Figure 11 depicts the calculated Raman spectra for the magnetic [E­(MIM)_2_]­[2FeCl_4_] system in the gauche and trans conformations. A characteristic peak can be observed at 596 cm^–1^ for the gauche conformer. In turn, the trans conformation is reflected at 644 cm^–1^. The anion substantially shifts the corresponding Raman frequencies compared to [E­(MIM)_2_]­[2Cl]. Again, the trans conformation appears to be more energetically favorable. This is most likely because the anion···anion electrostatic repulsion is minimized. The calculations of all chemical compositions show acceptable agreement with the experimental data provided in Figure 11.

**Figure 11:**
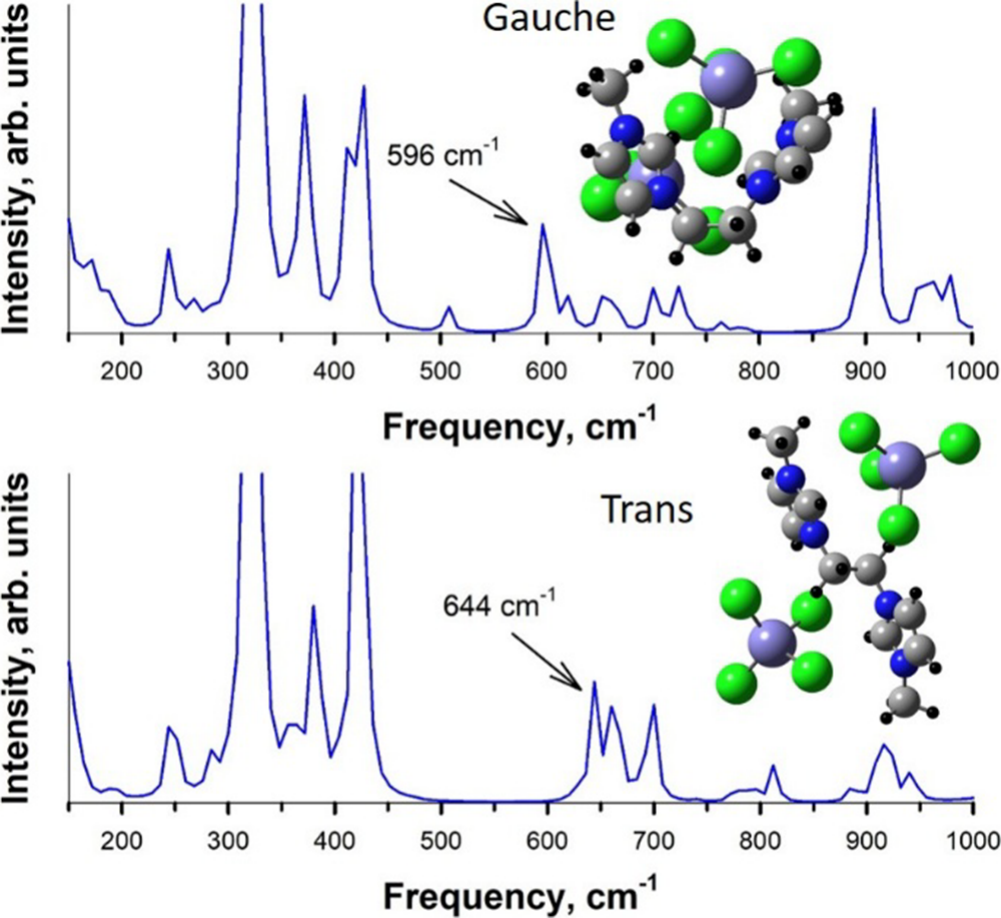
Raman spectra for [E­(MIM)_2_]­[2FeCl_4_] in the gauche conformation (top) and the trans conformation (bottom). The simulated geometries of the respective conformations are provided as insets.

### 3.3 Analysis of Physical Properties

Table 1 summarizes the physicochemical properties of ILs. Measurements of physical properties were conducted at the same temperature as the CO_2_ sorption and kinetic tests (40 °C) to better understand the behavior of the ILs at this temperature.

The density values followed a logical trend based on the molecular mass of the ILs in the following order: [E­(MIM)_2_]­[2FeCl_4_] > BMIM FeCl_4_ > [E­(MIM)_2_]­[2Cl] > BMIM Cl. This trend reflects the contribution of iron and chlorine atoms, which increase the density as anticipated.

The order of IL viscosity was observed as BMIM FeCl_4_ > [E­(MIM)_2_]­[2FeCl_4_] > [E­(MIM)_2_]­[2Cl]. The viscosity of BMIM Cl could not be measured because it existed in a semisolid state at 40 °C, rendering it incompatible with the available equipment. In general, DILs exhibited lower viscosity compared to MoILs. A correlation between viscosity and Raman spectra suggests that a lower gauche isomer conformation peak corresponds to reduced viscosity, potentially linked to interactions between the cation and anion.

Thermal stability of the ILs was assessed by using TGA and DTG curves (Figure 12). The values for *T*_Onset_, *T*_Max_, and *T*_End_, can be seen in Table 1 and Figure 12. The initial mass loss was less significant for more viscous ILs, likely due to reduced evaporation of physically adsorbed water from ambient humidity. This effect may be attributed to van der Waals forces and hydrogen bonding, which make more viscous ILs more hydrophobic than their less viscous counterparts.^56^ The second mass loss corresponds to ILs degradation. For MILs, third and fourth mass losses were observed, associated with the FeCl_4_^–^ anion, which presents greater thermal stability compared to the Cl^–^ anion.^57^

**Figure 12:**
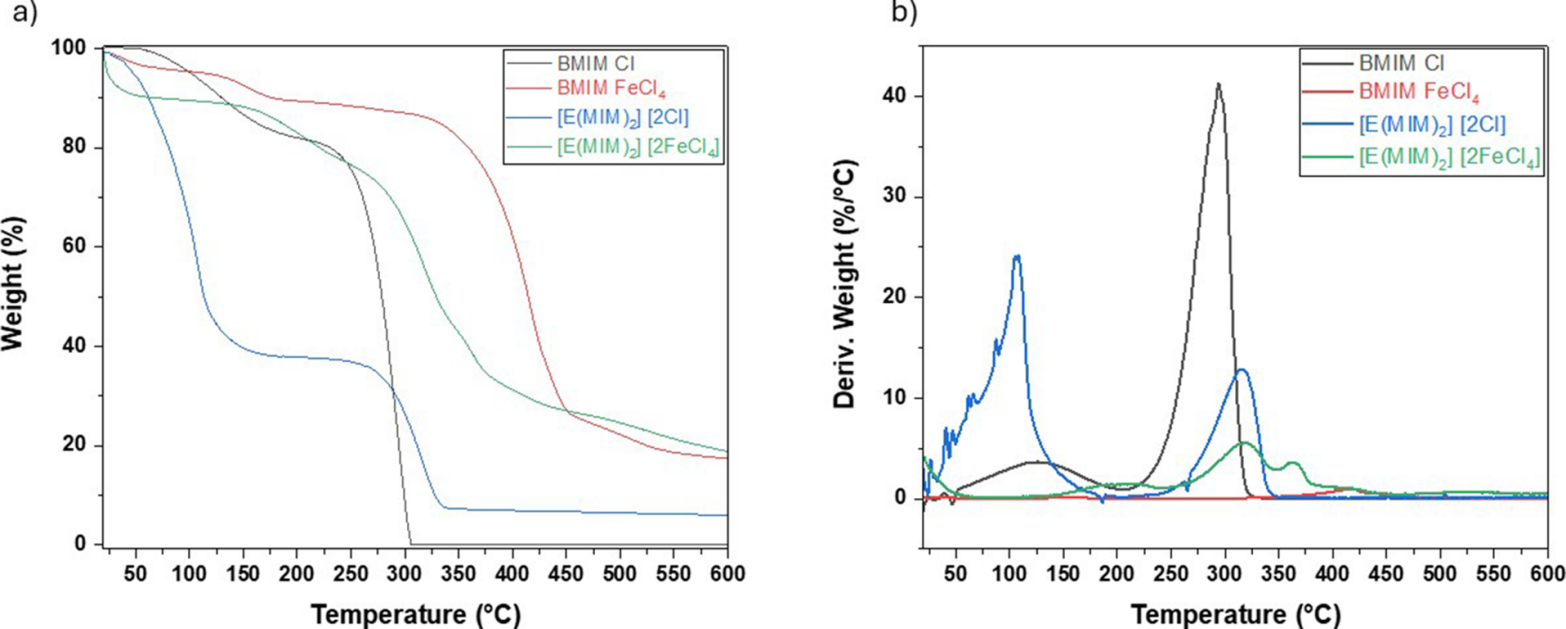
(a) Thermogravimetric analysis (TGA) and (b) first derivative (DTG) curves of the BMIM Cl, BMIM FeCl_4_, [E­(MIM)_2_]­[2Cl], and [E­(MIM)_2_]­[2FeCl_4_].

### 3.4 Studies of CO_2_ Capture, Kinetics, CO_2_/N_2_ Selectivity, and Recyclability

The kinetic behavior exhibited in Figure 13b, in which [E­(MIM)_2_]­[2FeCl_4_] demonstrates a higher initial CO_2_ sorption rate for approximately 6 min, followed by [E­(MIM)_2_]­[2Cl] exhibiting superior performance at later times, may be related. by the operation of disparate mechanisms throughout the process. In the initial stages, sorption is dominated by the affinity between CO_2_ and the active sites of the ionic liquid, being favored in [E­(MIM)_2_]­[2FeCl_4_], possibly due to the presence of the magnetic and highly polarizable anion [FeCl_4_]^−^, which intensifies electrostatic and quadrupole-ion interactions with the CO_2_ molecule, resulting in faster initial kinetics.^58–60^ However, as the system evolves, the diffusion of CO_2_ in the liquid volume becomes the limiting factor, a step strongly influenced by the viscosity of the medium. In this regime, [E­(MIM)_2_]­[2Cl], which possesses markedly reduced viscosity, provides less resistance to mass transport, thereby facilitating greater continuous accessibility to interaction sites and promoting more efficient sorption over time. Consequently, while [E­(MIM)_2_]­[2Cl] exhibits diminished initial kinetic rates, it attains an augmented final sorption capacity. This observation signifies a shift from a regime governed by initial interaction to one that is predominantly influenced by diffusion within the ionic liquid volume.^61–63^ Conversely, BMIM Cl exhibited no sorption capacity for up to 135 min due to its initial semisolid state at 40 °C. At the conclusion of the experiment, BMIM Cl had undergone a phase transition, transforming into a liquid with extremely high viscosity. In a similar manner, BMIM FeCl_4_, despite its high viscosity, initiated CO_2_ sorption at approximately 27 min after the commencement of the test.

**Figure 13:**
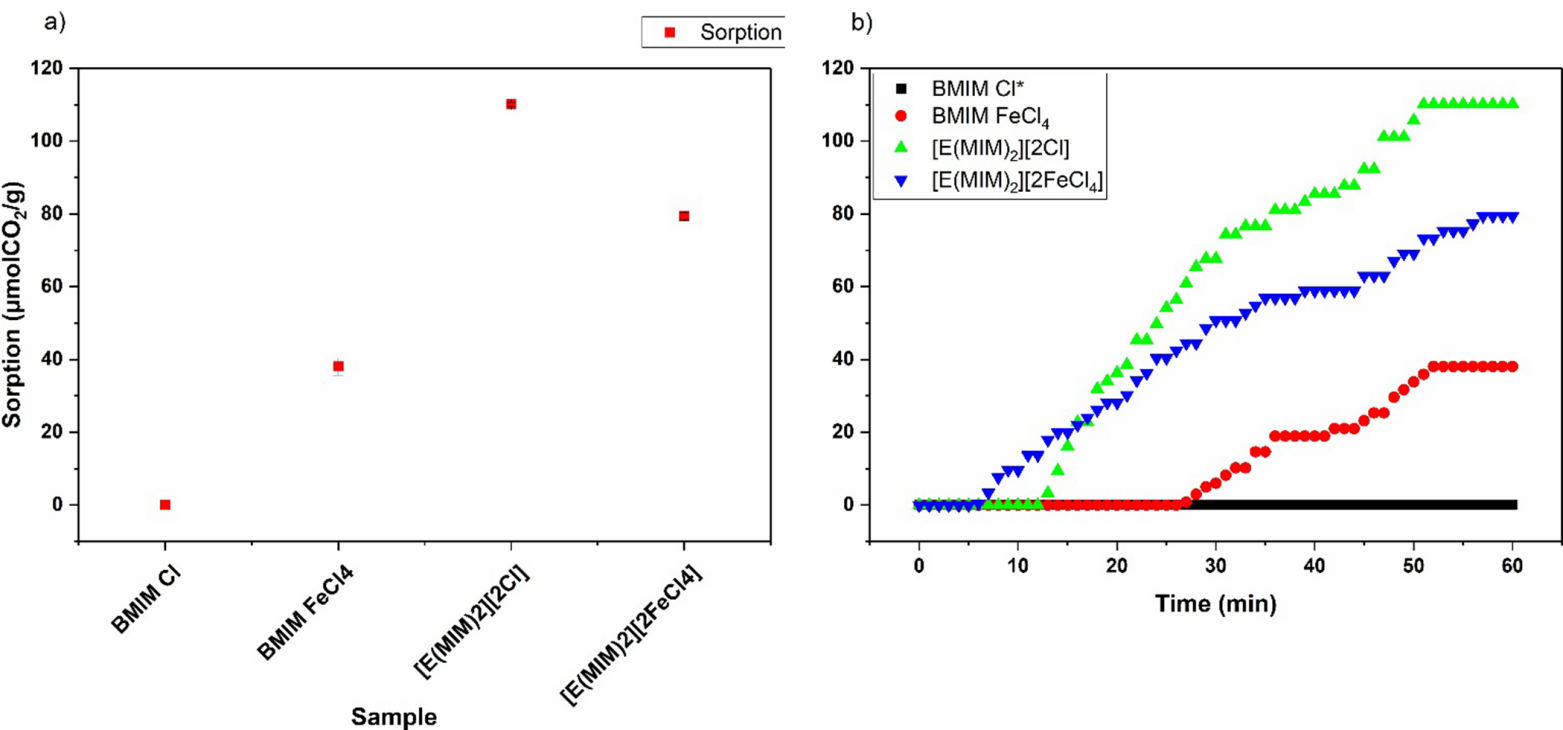
Test of (a) sorption and (b) kinetics of CO_2_ with BMIM Cl, BMIM FeCl_4_, [E­(MIM)_2_]­[2Cl], and [E­(MIM)_2_]­[2FeCl_4_].

The absorption of CO_2_ by ILs depends on its solubility, which is often said to be dominated by the strength of CO_2_’s interaction with anions.^64^ However, studies by Babarao et al., Gupta et al., and Sistla et al. point out that the CO_2_ solubility is primarily governed by cation–anion binding energy and secondarily by the CO_2_-anion binding energy. These studies suggest that weaker cation–anion interactions lead to greater CO_2_ solubility due to the formation of cavities that allow for CO_2_ to be accommodated. For example, fluoroalkyl anions are considered the most soluble for CO_2_ because they are weak Lewis bases that create more interstitial spaces in the IL network due to weak cation–anion interactions.^65–67^ Therefore, the conformational aspects of the ILs studied in this article indicate that samples with a greater trans conformation presence have a lower viscosity. This may be associated with weaker cation–anion interactions that improve CO_2_ absorption ability. Viscosity is a property governed by three factors: size, shape, and the interaction between the anion and the cation.^68^

In addition, the significantly higher sorption performance for DILs can be linked to the greater availability of active sites for interaction with CO_2_, when compared to conventional monocations, creating a greater number of cavities for gas allocation. A series of previous studies, most of them computational, point to this same aspect, which can be proven efficient in a practical way through this work.^18,64,69,70^

The effect of pressure on CO_2_ sorption by DIL [E­(me)_2_]­[2Cl] was investigated in the range of 1 to 10 bar, and the results are shown in Figure 14. An almost linear increase in the level of CO_2_ sorption can be observed with increasing pressure in the system. At a pressure of 1 bar, the solubility of CO_2_ was found to be 68.56 (±2.84) μmol/g, which increased to 110.20 (±1.61) μmol/g at 4 bar. This was followed by a pronounced increase at higher pressures, with values of 240.90 (±0.80) μmol/g at 7 bar and 367.35 (±5.42) μmol/g at 10 bar. This behavior is in accordance with Henry’s second law, which has been documented in the context of nonreactive ionic liquids.^71^

**Figure 14:**
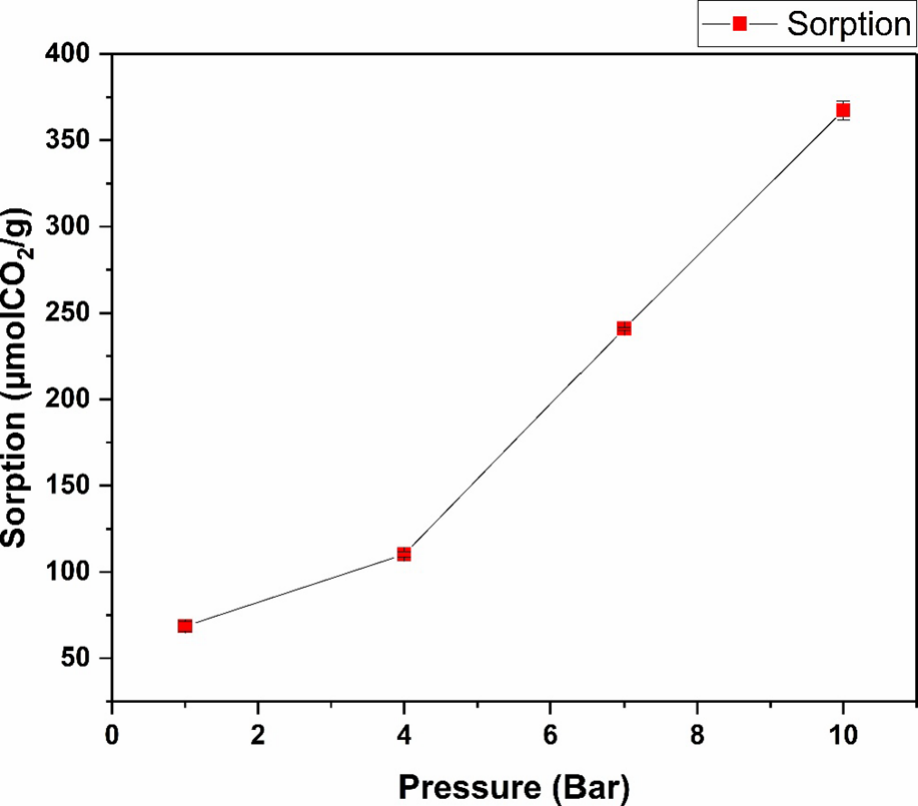
CO_2_ sorption by [E­(mim)_2_]­[2Cl] at 40 °C in the pressure range of 1–10 bar.

The CO_2_ sorption data obtained with [E­(me)_2_]­[2Cl] are compared to those of other DILs found in the literature (Table 2). At a pressure of 4 bar and a temperature of 40 °C, the CO_2_ sorption of [E­(min)_2_]­[2Cl] was 110.20 μmol/g, which is comparable to or superior to other DILs, such as PDBr and PDNTf_2_, conducted at 2 bar and 25 °C, and to [tetraEG­(min)_2_]­[Br]_2_, conducted at 1 bar and 70 °C.^72,73^ A favorable response to pressure can be highlighted, despite the use of a relatively small chloride anion that is less akin to that of CO_2_. The DILs 3OEt-Im, DABCO-B and phosphonium-based DILs with DOSS^–2^ anions are often attributed to increased free volume and greater affinity for CO_2_, but this is often at the expense of viscosities.^74,75^ The samples were subjected to testing under conditions of elevated pressure (10 and 5 bar) and temperate temperatures (25 °C), conditions that are more favorable for CO_2_ sorption. However, the results obtained with [E­(min)_2_]­[2Cl] are competitive despite its simple structure and the fact that it was tested under more adverse conditions.

**Table 2:** Comparison of Data Obtained with Literature Data on CO_2_ Solubility in Ionic Liquids

dicationic ionic liquids	temperature (°C)	pressure (bar)	CO_2_ solubility (μmol CO_2_/g)	refs
DABCO-B	25	10	193	^74^
3OEt-Im	25	10	433	^74^
[P_8,8,8_ C_6_P_8,8,8_]DOSS_2_	25	5	116.7	^75^
[P_8,8,8_ C_10_P_8,8,8_]DOSS_2_	25	5	121.2	^75^
PDBr	25	2	52.3	^72^
PDNTf_2_	25	2	95.4	^72^
[tetraEG(mim)_2_][Br]_2_	70	1	47.54	^73^
[E(MIM)_2_][2Cl]	40	4	110.20	This work

To simulate the environment of postcombustion systems, a sorption experiment was conducted using N_2_, the primary gaseous component in these processes.^76^ The goal was to evaluate the solubility of [E­(MIM)_2_]­[2Cl], which was previously identified as the most promising for CO_2_ capture under the proposed conditions. This test is essential to verify the solvent’s selectivity, as the presence of large proportions of N_2_ could compromise the performance of sorbent materials in a selective manner. After completing the tests, we observed that the ionic liquid did not significantly interact with N_2_ molecules under the established operating conditions, as illustrated in Figure 15. This result indicates that [E­(MIM)_2_]­[2Cl] has a high selectivity for CO_2_, making it a promising candidate for selective gas separation processes, particularly in carbon emission mitigation.

**Figure 15:**
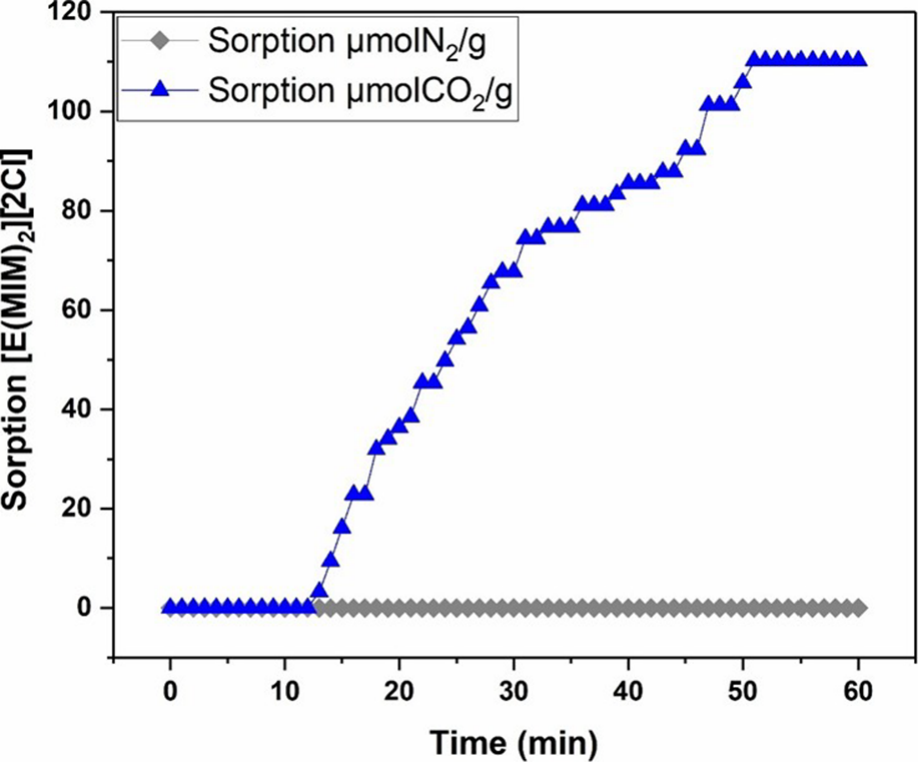
Solubility of IL [E­(MIM)_2_]­[2Cl] in CO_2_ and N_2_.

The recyclability test of [E­(MIM)_2_]­[2Cl] was performed to evaluate the stability of the CO_2_ capture. The ionic liquid was subjected to five sorption cycles over a period of 60 min, at 40 °C and 4 bar at equilibrium. Desorption was performed by reducing the pressure at 40 °C over a period of 1 h. As can be seen in Figure 16, [E­(MIM)_2_]­[2Cl] maintained good stability, with an average sorption capacity of 110.82 (±1.82) μmol CO_2_/g. This indicates that the material is highly recyclable after five sorption cycles under these conditions.

**Figure 16:**
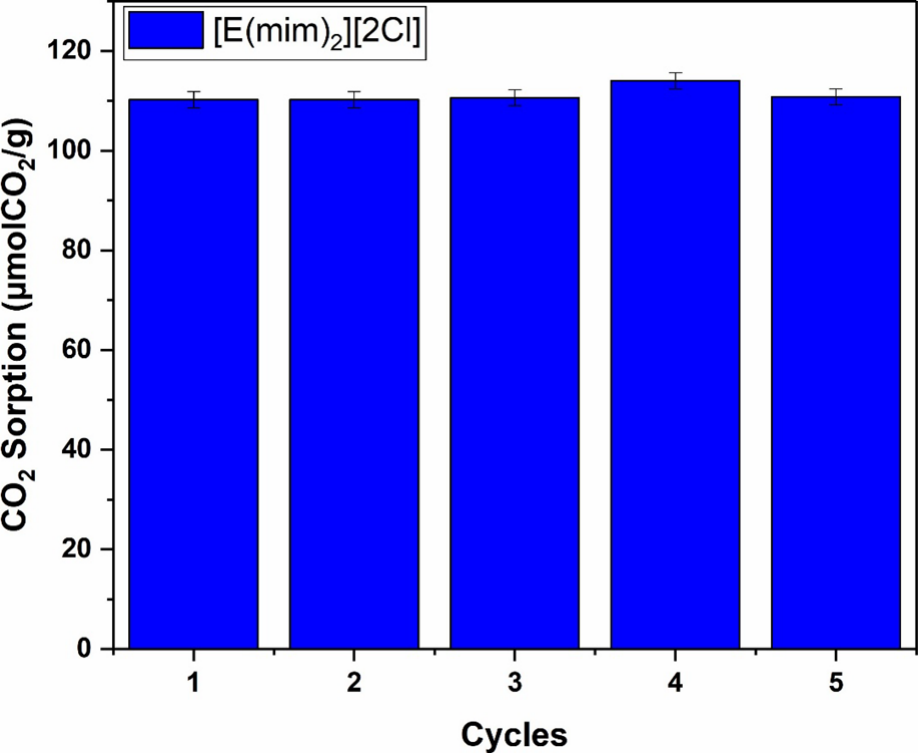
Recyclability test of [E­(MIM)_2_]­[2Cl].

## 4 Conclusions

The experiments revealed that cation–anion interactions play a pivotal role in determining the properties of ILs. Raman spectroscopy indicated that conformational isomers significantly impact the viscosity and melting behavior. ILs with a predominance of trans conformations exhibited lower viscosity and were stable in a liquid form at room temperature, whereas the presence of the gauche conformation suggested interactions associated with larger ionic structures. The data obtained by experimental spectra were corroborated by the computer simulation performed, which presented an energetically more favorable conformation for the compounds. Regarding CO_2_ uptake and kinetics, DILs demonstrated superior performance in both aspects. This enhanced performance may be attributed to the increased availability of active sites and the reduced viscosity of the DILs, which resulted in enhanced CO_2_ diffusion. This behavior can also be attributed to the diminished cation–anion interaction force in DILs, a property that enables more effective interactions between CO_2_ and the ILs. The [E­(MIM)_2_]­[2FeCl_4_] exhibited faster initial kinetics, whereas [E­(MIM)_2_]­[2Cl] achieved higher CO_2_ absorption due to its significantly lower viscosity. This lower viscosity resulted in reduced resistance to mass transport, greater continuous accessibility to interaction sites, and more efficient sorption over time. Furthermore, [E­(MIM)_2_]­[2Cl], which exhibited optimal CO_2_ sorption performance under the proposed conditions, was evaluated in an N_2_ atmosphere under identical conditions. No interaction with N_2_ was observed, thereby suggesting a particular affinity for the compounds of CO_2_. During the recyclability assessment, the ionic liquid demonstrated stability after undergoing five cycles of sorption and desorption.
